# CE–MS-based urinary biomarkers to distinguish non-significant from significant prostate cancer

**DOI:** 10.1038/s41416-019-0472-z

**Published:** 2019-05-16

**Authors:** Maria Frantzi, Enrique Gomez Gomez, Ana Blanca Pedregosa, José Valero Rosa, Agnieszka Latosinska, Zoran Culig, Axel S. Merseburger, Raul M. Luque, María José Requena Tapia, Harald Mischak, Julia Carrasco Valiente

**Affiliations:** 1 Mosaiques diagnostics GmbH, Hannover, Germany; 2 Urology Department, Reina Sofía University Hospital, Cordoba, Spain; 3 Maimonides Institute of Biomedical Research of Cordoba (IMIBIC), Cordoba, Spain; 4 Department of Cell Biology, Physiology and Immunology, University of Cordoba (UCO), Cordoba, Spain; 5 Division of Experimental Urology, Department of Urology, Medical University of Innsbruck, Innsbruck, Austria; 6 Department of Urology, University Clinic of Schleswig-Holstein, Campus Lübeck, Lübeck, Germany; 7 CIBER Physiopathology of Obesity and Nutrition (CIBERobn), Madrid, Spain; 8 Agrifood Campus of International Excellence (CeiA3), Cordoba, Spain

**Keywords:** Prostate cancer, Diagnostic markers, Proteomic analysis

## Abstract

**Background:**

Prostate cancer progresses slowly when present in low risk forms but can be lethal when it progresses to metastatic disease. A non-invasive test that can detect significant prostate cancer is needed to guide patient management.

**Methods:**

Capillary electrophoresis/mass spectrometry has been employed to identify urinary peptides that may accurately detect significant prostate cancer. Urine samples from 823 patients with PSA (<15 ng/ml) were collected prior to biopsy. A case–control comparison was performed in a training set of 543 patients (*n*_Sig_ = 98; *n*_non-Sig_ = 445) and a validation set of 280 patients (*n*_Sig_ = 48, *n*_non-Sig_ = 232). Totally, 19 significant peptides were subsequently combined by a support vector machine algorithm.

**Results:**

Independent validation of the 19-biomarker model in 280 patients resulted in a 90% sensitivity and 59% specificity, with an AUC of 0.81, outperforming PSA (AUC = 0.58) and the ERSPC-3/4 risk calculator (AUC = 0.69) in the validation set.

**Conclusions:**

This multi-parametric model holds promise to improve the current diagnosis of significant prostate cancer. This test as a guide to biopsy could help to decrease the number of biopsies and guide intervention. Nevertheless, further prospective validation in an external clinical cohort is required to assess the exact performance characteristics.

## Background

Prostate cancer (PCa) is ranked as the second most frequently diagnosed cancer in men,^1^ and the most frequent non-skin cancer in developed countries.^2^ Although PCa is diagnosed in 15–20% of men, the lifetime risk of death due to PCa is very low (3%),^3^ mainly because low-risk forms progress slowly and the disease is well treatable in early stages. PCa diagnosis is currently mostly based on serum prostate-specific antigen (PSA) testing, digital rectal examination (DRE) and confirmed by a multi-core prostatic biopsy.^4^ Multiple factors not related to prostate malignancy may affect the level of blood PSA [inflammation, infection or presence of benign prostate hyperplasia (BPH)]. Therefore, PSA lacks specificity particularly in the intermediate range, with only 22–27% of those patients with PSA between 4–10 ng/ml to be positively confirmed with PCa after biopsy.^5^ In addition, PSA screening and multicore biopsy have increased the detection rate of small, localised, well-differentiated PCa,^6^ resulting in over-diagnosis and over-treatment.^6–9^ For those patients presenting with an indolent or clinically non-significant cancer (Gleason score (GS) < 7),^10^ immediate treatment may not be beneficial and ideal management may be a conservative approach, such as active surveillance (AS).^11^ Management of patients with non-significant PCa currently relies on repeated biopsies, series of PSA measurements and DRE, while the uncertainty to properly assess PCa imposes a significant social and economic burden on patients and health insurances because of the side effects and treatment costs.^12^ For these reasons, better stratification of the risk for significant PCa (Sig PCa) appears beneficial to guide patient management.

Aimed at improving on the current discrimination of Sig PCa by non-invasive means, capillary-electrophoresis coupled to mass spectrometry (CE–MS) was employed to identify peptides specific for PCa in urine samples from patients with clinically significant and non-significant PCa. Urine was selected, as it presents several advantages over blood or tissue, among others: easy, non-invasive repeated sampling, effortless availability and high stability of the proteome. Although several candidate biomarkers have been described,^13–15^ the currently available single biomarkers lack diagnostic accuracy for routine clinical application. At the same time, the high biological variability of PCa suggests that a combination of clearly defined, -omics derived biomarkers, rather than a single biomarker, may provide higher accuracy to detect cancer.^16–18^ In this study, we aimed to establish a biomarker model to detect Sig PCa.

## Methods

### Study population and design

A case–control study was performed on patients who underwent a transrectal ultrasound (TRUS)-guided prostate biopsy from January 2013 to July 2015 in the Urology department, Reina Sofia Hospital, Cordoba, Spain, as part of the ONCOVER project. Ethical approval was obtained by the Reina Sofia Hospital Research Ethics Committee and informed consent was obtained from all participants for the project. ONCOVER cohort included patients who attended the urology clinic of Reina Sofia Hospital with a recommendation for a prostate biopsy according to clinical practice.^19^ Patients provided a urine sample and underwent blood testing just before undergoing a prostate biopsy. Recommendations for biopsy indication were: suspicious findings on DRE, PSA > 10 ng/mL, or PSA 3–10 ng/mL if free PSA ratio was low (usually, <25–30%), and in patients with previous biopsies, a persistently suspicious indication of PCa (persistently elevated PSA, suspicious DRE, etc.). For transrectal prostate biopsy, 12 cores were obtained from patients undergoing the first biopsy procedure, and a minimum of 16 biopsy cores for those who had a previous biopsy. For this analysis, 823 PCa patients were included according to the following criteria: (a) PSA level <15 ng/mL on the day of the biopsy and (b) no previous diagnoses of PCa. For all 823 patients, complete records for all the main variables were available, including PSA, DRE, number of previous biopsies, 5-alpha-reductase inhibitor intake and pathology results. Information on prostate volume was additionally retrieved for 721 patients, based on the measurements that had been performed with TRUS during the biopsy. Because of missing data for 102 patients, and in order to avoid introducing any selection bias, for this analysis prostate volume was not included in the nomogram analysis, but only for comparison purposes (i.e., biomarkers compared to prostate volume). Patients treated with 5-alpha-reductase inhibitors for urinary symptoms were also included in the study, but excluded from the analysis for the comparison with PSA, as treatment with 5-alpha-reductase inhibitors is expected to affect PSA levels. All biopsy specimens were analysed by a urologic pathologist according to International Society of Urological Pathology 2005 modified criteria.^20^ Clinical and laboratory data, including among others: age, PSA level (on the day of biopsy), the results of DRE, number of previous biopsies, prior treatment with 5-alpha-reductase inhibitors, prostate volume by TRUS, urinary creatinine and pathology results were collected and presented in the Supplementary Table 1. A score based on the risk calculator of the European Randomised Study of Screening for Prostate Cancer (ERSPC) was calculated (http://www.prostatecancer-riskcalculator.com/seven-prostate-cancer-risk calculators). The formulas that were utilised in this study, were ERSPC- 3, for those patients during initial biopsy, and ERSPC- 4, for patients during repeated biopsy. For the above estimates, the variables that are considered are PSA and DRE and the result of previous biopsy for those patients who underwent (biopsy before (ERSPC-4). GS was used in this study to discriminate Sig PCa (GS ≥ 7) from non-Sig PCa.

### MS analysis

CE–MS analysis was performed for the 823 urine samples, following the previously established protocols for samples preparation and data acquisition, previously described in detail.^21^ In brief, sample preparation was performed by diluting 700 µl urine aliquots from the urine collected from patients prior to the prostate biopsy, with two volumes (1.4 ml) alkaline buffer containing 2 M urea, 10 mM NH_4_OH and 0.02% sodium dodecyl sulphate (pH 10.5). Subsequently, the samples were filtered by Centrisart ultracentrifugation filters (Sartorius, Göttingen, Germany) to retain proteins/polypeptides below 20 kDa and were subsequently desalted over PD-10 columns (GE Healthcare, Munich, Germany). The peptide extracts were lyophilised and resuspended in high-performance liquid-chromatography (LC) grade water. CE–MS analysis and data processing were performed according to ISO13485 standards yielding quality controlled urinary data sets.^21^ Mass spectral ion peaks representing identical molecules at different charge states were deconvoluted into single masses using MosaiquesVisu software.^22,23^ The peak list characterises each peptide by its molecular mass (kDa), normalised migration time (min) and normalised signal intensity (AU).^22,23^ Normalisation of the CE–MS data were based on twenty nine collagen fragments that are generally not affected by disease and serve as internal standards.^24^ After normalisation, all proteomics datasets were deposited, matched, and annotated in a Microsoft SQL database and used as input in the presented study. Transformation of the data (log-transformation) was performed prior to the statistical analysis, as previously described.^25^

### Peptide sequencing and matching

Matching of the amino acid sequences with the CE–MS acquired ion peaks was based on mass correlation between CE–MS and LC-tandem MS analysis. The amino acid sequence was determined by MS/MS analysis using either a PACE CE or a Dionex Ultimate 3000 RSLS nanoflow system (Dionex, Camberly UK) coupled to an Orbitrap Velos instrument (Thermo Scientific), as previously described.^26^ Protein matching and data analysis was based on Proteome Discoverer 1.2 (activation type: HCD; precursor mass tolerance: 5 ppm; fragment mass tolerance: 0.05 Da). No fixed modifications were selected, oxidation of methionine and proline were selected as variable modifications. The data were searched against the UniProt human database^27^ without enzyme specificity. Further validation of the obtained peptide identifications is based on the assessment of the peptide charge at the working pH of 2.2 and the CE-migration time results.^28^

### Statistical analysis

A case–control statistical comparison was performed to detect potentially Sig PCa biomarkers. The datasets were grouped into: (a) a case set of clinically Sig PCa (*n*_Sig_ = 146), including PCa patients with high-risk PCa (GS ≥ 7) and (b) a control set including clinically non-significant PCa (low-risk PCa; GS = 6) along with patients presenting with other aetiologies (*n* = 677). The groups were further divided into a discovery (*n*_Sig_ = 98 cases of Sig PCa; *n*_non-Sig_ = 445 controls) and validation set (*n*_Sig_ = 48 cases of Sig PCa, *n*_non-Sig_ = 232 controls), according to the ‘2/3–1/3 rule’, as previously described.^16^ Random sampling guarantees that each group/class is properly represented in all data subsets. Based on the literature,^29^ this commonly used strategy of allocating two-third of cases for training is close to optimal for large sized datasets (*n* ≥ 100) with strong signals (i.e., >85% full dataset accuracy).^29^

Further statistical analysis was performed to identify potential bias, considering the clinical data shown in Table 1. Mann–Whitney non-parametric test was used to investigate statistically significant differences between the two groups for continuous variables and chi-squared test for categorical variables, respectively. The urinary CE–MS profiles were compared for differences at the individual peptide excretion levels by applying the Wilcoxon rank sum test.^25^ A frequency threshold of 70% in at least one of the two groups was applied. To increase the validity of the statistical approach, permutation analysis was performed by randomly excluding 30% of the samples and repeated five times. Statistical correction of the estimated *p* values for multivariate testing was performed based on the Benjamini–Hochberg method.^30^ Only the peptides significant (*p* < 0.05) in all five permutation analyses were considered for further analysis.

**Table 1:** Clinical and biochemical variables for the patients grouped into the discovery and validation set

Baseline characteristics	Discovery phase (*n* = 543)	Validation phase (*n* = 280)	*p* Value discovery vs. validation	Group 1: Non-significant PCa/Controls (*n* = 677)	Group 2: Significant PCa (*n* = 146)	*p* Value group 1 vs. group 2
Median age (IQR; y)	64.0 (11.0)	63.5 (12.0)	0.2947^a^	63.0 (11.5)	68.0 (10.3)	<0.0001^a^
PSA median (IQR; ng/ml)	5.4 (3.6)	5.0 (3.1)	0.2060 ^a^	5.1 (3.3)	6.1 (4.1)	0.0013^a^
Digital rectal examination (Pos/Neg)	104/439	40/240	0.0576^b^	94/583	50/96	0.1453^b^
Previous biopsies (Y/N)	139/404	69/ 211	0.9895^b^	187/480	21/125	0.0007^b^
Median number of previous biopsies (IQR)	1 (1)	1 (0)	0.6366^a^	1 (1)	1 (0)	0.007^a^
Prostate volume (IQR; ml)	35.0 (25.0; *n* = 481)	36.0 (18.4; *n* = 240)	0.6701^a^	37.4 (23; *n* = 594)	28.0 (18; *n* = 127**)**	<0.0001^a^
PSA density (IQR; ng/ml^2^)	0.14 (0.11; *n* = 481)	0.14 (0.10; *n* = 240)	0.3156^a^	0.20 (0.09; *n* = 594)	0.13 (0.15; *n* = 127)	<0.0001^a^
5α-reductase treatment (Y/N)	18/ 524	6/ 274	0.4640^b^	22/655	2/144	0.2219^b^
Median urinary creatinine (IQR; mmol/L)	7.6 (4.7)	8.3 (4.9)	0.0875^a^	7.9 (4.7)	7.8 (4.7)	0.5319^a^
Significant PCa	98 (18.0%)	48 (17.1%)	0.9345^b^			
Non-significant PCa	445 (82.0%)	232 (82.9%)	0.8532^b^			
*Disease pathology—significant PCa*						
Gleason 3 + 4/4 + 3	63 (64.3%)/20 (20.4%)	32 (66.6%)/9 (18.8%)	0.9290^b^/0.9945^b^			
Gleason 8	9 (9.2%)	5 (10.4%)	0.9459^b^			
Gleason≥9	6 (6.1%)	2 (4.2%)	0.9308^b^			
*Disease pathology—non-significant PCa*						
Gleason 6	99 (22.1%)	32 (13.8%)	0.0126^b^			
*Benign—non-PCa aetiologies*						
Benign prostatic hyperplasia; BPH	307 (69.1%)	175 (75.4%)	0.1033^b^			
Prostatic intraepithelial neoplasia; PIN	22 (5.0%)	12 (5.2%)	0.9425^b^			
Atypical small acinar proliferation; ASAP	17 (3.8%)	13 (5.6%)	0.3766^b^			

^a^Mann–Whitney test

^b^Chi-squared test

*IQR* interquartile range, *N* not received, *Neg* negative, *PCa* prostate cancer, *Pos* positive, *Y* received

### Optimisation of the SVM-based biomarker model

The urinary peptide-based classifier was developed in the training set, using MosaCluster (version 1.7.0), a support vector machine (SVM)-based software. The classifier was optimised based on the shortlisted PCa specific biomarkers with each biomarker representing one dimension in the n-dimensional parameter space.^17^ In additiony, the cut-off was established based on the discovery set. In the independent validation, the sensitivity and specificity estimates for the SVM-based peptide marker pattern were calculated based on the number of correctly classified samples. The receiver operating characteristic (ROC) plots and the respective confidence intervals (95% CI) were based on exact binomial calculations and were calculated in MedCalc 12.7.5.0 (Mariakerke, Belgium). Area under the curve (AUC) values were then compared using DeLong tests. Statistical comparisons of the classification scores in the validation cohort were performed by the Kruskal–Wallis rank sum test using MedCalc. To address the potential clinical utility of the models, we performed decision curve analysis, as proposed by Vickers and Elkin.^31^ This method has the advantage of not requiring the specification of the relative cost for false-positives and false-negatives, defining a net benefit as a function of the decision threshold at which one would consider obtaining a biopsy. For the analysis MedCalc 12.7.5.0 (Mariakerke, Belgium) and R version 3.2.3 were used.

## Results

### Study cohort for patients with clinically significant and non-significant PCa

Proteomics profiling data were acquired from 823 patients suspicious for PCa. Out of those, 677 (82.3%) presented with non-significant PCa (GS = 6), benign or atypical conditions (control group) and 146 (17.7%) were included in the case group due to presence of Sig PCa. Men with Sig PCa were significantly older [median age = 68; interquartile range (IQR) = 10.3] compared to men from control group (median age = 63; IQR = 11.5; *p* *<* 0.0001). In addition, patients from the control group had significantly lower PSA levels (median = 5.1 ng/ml; IQR = 3.3) compared to those from case group [median = 6.1 ng/ml; IQR = 4.1; *p* *=* 0.0013]. Within the control group, 480 (70.9%) did not undergo any previous negative biopsy, while for patients with Sig PCa, the respective proportion was 85.6% (*n* = 125); (*p* *=* 0.0007). The clinical characteristics along with the sample distribution are presented in the Table 1.

### Development of a biomarker model based on CE–MS urinary peptide profiling

**Fig. 1:**
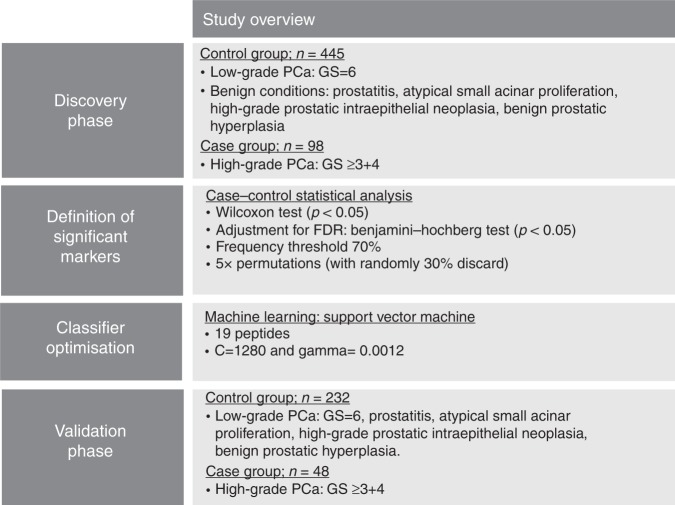
Schematic representation of the study design and the analytical workflow for the development of urine CE–MS-based biomarker panel

**Fig. 2:**
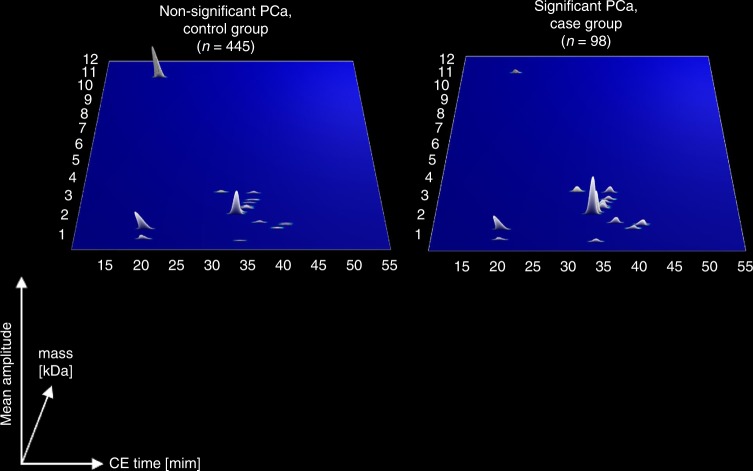
Compiled average urinary profiling signatures of the patients with significant and non-significant PCa. The molecular mass (0.1–12 kDa) is shown on a logarithmic scale and is plotted against normalised migration time (15–55 min). Signal intensity is encoded by peak height and colour

For the identification of CE–MS specific biomarkers, a case–control comparison was performed in the discovery set of 543 patients, schematically depicted in Fig. 1. The comparison enabled the identification of 19 peptides displaying statistically significant differences in their distribution between patients with Sig PCa compared to the control group (Supplementary Table 2). The graphical depiction of the compiled urinary profiling signatures is comparatively presented in Fig. 2. Using the 19 statistically altered peptide markers an SVM machine learning algorithm was adopted and optimise to develop a classifier (Fig. 1).

### Independent validation of the SVM-based biomarker model

**Fig. 3:**
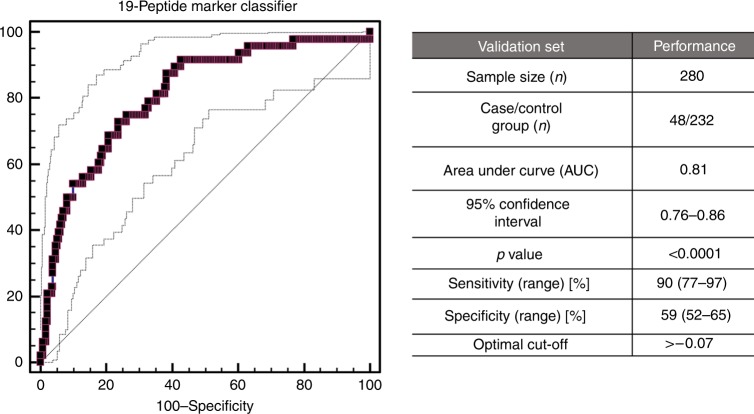
Receiver operating characteristics (ROC) analysis performed in the independent validation cohort, displaying the performance of the 19-biomarker panel for discriminating the case group (*n*_Sig_ = 48) from the control group (*n*_non-Sig_ = 232). ROC characteristics, such as area under the curve (AUC), 95% confidence intervals (CI), and *p* value are provided for the classification of Sig PCa patients

**Fig. 4:**
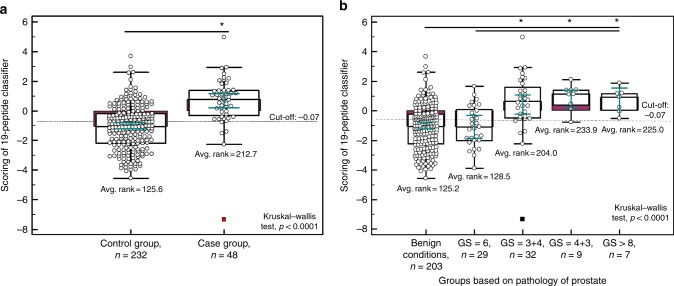
**a** Classification scores, presented in Box-and-Whisker plots grouped according to the case group (*n*_Sig_ = 48) and control group (*n*_non-Sig_ = 232). **b** Classification scores displaying the level of discrimination across the different Gleason score. A post hoc rank-test was performed using Kruskal–Wallis test. *p < 0.05

Validation of the 19-biomarker model in the independent set (*n* = 280), in line with the recommendations for biomarker identification and reporting in clinical proteomics,^25^ resulted in an overall AUC value of 0.81 ranged from 0.76 to 0.86 (95% CI: *p* *<* 0.0001). Fig. 3 presents the ROC curve, which at the pre-defined cut-off of −0.07 resulted in sensitivity levels of 90% (77–97; 95% CI) and specificity of 59% (52–65; 95% CI), respectively. Additional statistical analysis was performed, by application of a post hoc rank sum test to compare the scores between the case and control groups. As depicted in Fig. 4a, the classification of each group differs at the significance level of *p* *<* 0.0001. Moreover, as shown in Fig. 4b, there is a gradual increase in the 19-biomarker model score, as GS increases, while a significant difference is observed between the 19-biomarker model scores of GS 6 tumours and GS ≥ 7 (*p* *<* 0.0001).

### Comparative analysis of the 19-biomarker model with clinical parameters

**Fig. 5:**
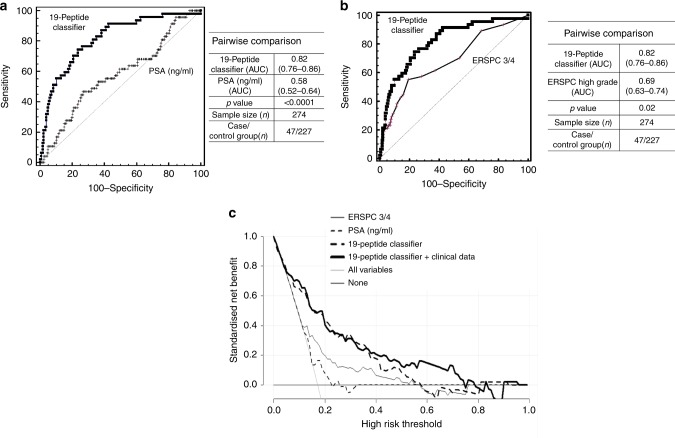
**a** Comparative analysis depicted by receiver operating characteristics (ROC) curves for the 19-biomarker panel and the PSA levels (*n* = 274). **b** Added value of the 19-biomarker panel over ERSPC -3/4 for high risk (Gleason ≥ 7) (*n* = 274; six patients from the validation set were excluded as previously treated with 5 alpha reductase inhibitors). **c** Results of the decision curve analysis. The net benefit for the prediction of Sig PCa on biopsy is shown, by using the different models as a function of the risk threshold, compared to the benefits of strategies for treating all patients (grey thin line) and treating none (grey thick line)

A direct comparison of the 19-biomarker model with PSA was performed in the validation set. Of note, out of 280 patients, 6 patients had received previous treatment with 5-alpha-reductase inhibitors, therefore for the comparative analysis only 274 patients were considered. As depicted in Fig. 5a, the multi-peptide model significantly outperformed the PSA testing with the AUC values at 0.82 and 0.58, respectively (*p* < 0.0001). For those patients where clinical records on prostate volume were available (*n* = 240), an additional comparison between the 19-biomarker model and the prostate volume was performed, indicating a significantly better accuracy for the 19-biomarker model (AUC of 0.81) compared to prostate volume (AUC of 0.64; *p* = 0.0103). Moreover, logistic regression analysis was performed for the available clinical variables to assess the potential significant predictive value of each of those in the discrimination of Sig PCa. The included clinical parameters were: (a) the result of DRE, (b) presence of previous biopsy, (c) the number of previous biopsies, (d) prostate volume and (e) age. Based on the statistical comparison significant contribution to the outcome is revealed for age (odds ratio of 1.1, *p* = 0.0366), PSA (odds ratio of 1.2, *p* = 0.0162) and the 19-biomarker model (odds ratio of 2.2, *p* < 0.0001), while the presence and number of previous biopsies, prostate volume and the result of DRE were not significant predictors of Sig PCa. Combination of the significant variables (19-biomarker model, PSA and age) into a nomogram through the regression equation, resulted in an improved AUC value of 0.83, although not statistically significant (*p* = 0.4344) compared to the 19-biomarker model alone. In order to investigate if the 19-peptide classifier can present an added value over the current state-of-the-art, the SVM-based score from the 19-biomarker model was further compared with the estimates of the ERSPC risk calculator for detecting high risk PCa (ERSPC—3/4), as presented in Fig. 5b. The 19-peptide classifier showed significantly better performance (AUC = 0.82; *p* = 0.02) compared to the ERSPC estimates (AUC = 0.69). To assess the clinical benefit of the 19-biomarker model, a decision curve analysis was additionally performed. Based on the net plotting against the threshold probabilities for the comparisons between the 19-biomarker model alone and with clinical variables (PSA, age), PSA and ERSPC estimates, there is a clear benefit of the biomarker model, particularly in the lower range of the risk thresholds (Fig. 5c).

### Sequencing of peptide biomarkers

Among the 19 peptide biomarkers, sequences could be obtained for 17, while 2 peptides could not be sequenced. The majority (14/17) were originated from various collagens. Peptide fragments originating from alpha-1 collagen of types (I), (XI), (XVII), (XXI) and alpha-2 type (I), (V), (IX), were most prominent and fragments of collagen type (VIII) chain were also identified. All the collagen peptide fragments are of increased abundance in the Sig PCa cases, apart from collagen alpha-1 (XVII) chain and collagen alpha-1(XXI), which are presented with decreased abundance. Interestingly, among the collagen fragments, a unique motif (pGP) is very prominent. The three remaining peptide markers were a fragment of protein phosphatase 1 regulatory subunit 3A, which was identified with decreased abundance and fractalkine or chemokine (C-X3-C motif) ligand 1 and Semaphorin-7A, both upregulated in the group of patients with Sig PCa.

## Discussion

Insignificant PCa are slowly progressing forms that may be better managed conservatively without immediate treatment. Nevertheless, as insignificant forms can progress to significant cancer, frequent monitoring is required to timely and accurately detect the progression. Currently, routine monitoring is based on either PSA, although associated low accuracy, or invasive biopsies.^32^ More accurate non-invasive biomarkers are required to improve on the discrimination of Sig PCa. In this study, a biomarker model based on urinary peptides was established and validated in 823 patients suspicious for presence of PCa. This peptide panel enables the discrimination of non-significant PCa from clinically significant forms with high sensitivity and moderate specificity. The lower specificity is mostly attributed to the misclassification of clinically non-significant PCa (mainly GS of 6) as clinically significant forms. The clinical consequence of this observation can be weighted as acceptable, since patients with a positive score based on the 19-biomarker model would further undergo biopsy to rule out the presence of significant cancer.

The 19-biomarker model performs significantly better, when compared to PSA levels and also, when compared to the ERSPC risk calculator, demonstrating an added value of the biomarkers. Comparison with other clinical variables was also performed indicating a significant improvement of the 19-biomarker model, although particularly for prostate volume, missing data for 34 patients from the validation set do compromise the statistical power. An additional decision curve analysis was performed to assess the clinical benefit of the 19-biomarker model, in comparison with the current clinical standards, PSA and ERSPC calculator, demonstrating an improved net benefit of the 19-biomarker model, particularly in the low range of risk threshold.

Nowadays, several biomarkers have been tested in order to discriminate Sig PCa, such as 4K score test, PHI, PCA3, SelectMDx).^33^ A direct comparison with those markers, was unfortunately not possible in the context of the presented study, as paired data were not available (as different cohorts and approximations were performed). However, the initial results shown in this study with an AUC higher than 0.80, is within the range of 0.74–0.90 which is shown by other biomarkers^34,35^ and clearly justify implementation of this approach in a future investigative setting. In line with this and in order to facilitate comparisons, an additional prospective validation study design is planned, similar to other studies, such as the step approximation of the STHLM3 study, which was able to identify up to 21% of Sig PCa in patients with a PSA between 1 and 3 ng/ml.^35^ In the prospective evaluation, inclusion of multiparametric magnetic resonance imaging is planned, as it has demonstrated an added value in the diagnostic approximation for Sig PCa with a high NPV, improving the detection of Sig PCa.^36,37^

Regarding the biomarker identity, sequences could be obtained from 17 of the 19 peptide markers, most of them derived from collagen origin and being in increased abundance. Collagen fragments represent the majority of urinary peptides, even in healthy individuals.^18^ The increase in specific collagen fragments may depict extracellular matrix rearrangements, associated with tumour invasion and resulting in proteolytic products, which are subsequently excreted in urine. Previous studies,^18,38^ reporting on CE–MS based biomarkers for detection of PCa (for discrimination of PCa patients from those without malignancy), also identified collagen fragments as being increased in abundance in cancer patients.^18,38^ In the present study, a slightly different clinical design was followed, as the aim was to discriminate in patients that had PCa, those presenting with significant cancer from those with non-significant cancer. In the study by Theodorescu et al.,^18^ four sequences out of twelve could be obtained, with one biomarker common in both studies: a fragment of Collagen alpha-1(I) chain. The other three biomarkers described by Theodorescu et al.^18^ were not identified as significantly altered in this study, while an enrichment was observed for other sequences belonging to collagen alpha-1 and collagen alpha-2 chains, protein phosphatase 1 regulatory subunit 3A and fractalkine. The observed differences are attributed in part to the advancements of the technology enabling a better sequence coverage, but also the different clinical context, which in this study was the identification of differentially abundant cancer biomarkers between two cancer forms. A pGP motif was present in most of the collagen sequences. The pGP motif is a chemoattractant derived from proteolytic cleavage of collagen by matrix metalloproteinases. pGP motif binds to (C–X–C motif) receptors and is thus associated with neutrophil attraction in inflamed tissues.^39^ In addition, protein phosphatase 1 regulatory subunit 3A, which is considered as a tumour suppressing molecule was identified with decreased abundance.^40^ Overall, the observations at the urinary peptides of the patients with Sig PCa, depict features of cancer progression and tumour related inflammation.

The specific clinical impact of the non-invasive biomarker model would primarily be to guide patient management and reduce the number of invasive biopsies. As such, high sensitivity is required, for correct detection of significant PCa. In view of a positive test, the treating physician is alerted to perform a more thorough investigation, improving the overall accuracy in detection of Sig PCa. Lower specificity would result in more misclassifications of non-significant PCa as potentially significant, and as a consequence prostate biopsy to rule out significant cancer. Therefore, a false positive result will be clarified upon biopsy.

These encouraging results should be interpreted considering the limitations of the study: Firstly, although the use of TRUS biopsy for PCa diagnosis suffers from random error and false negative results in comparison with trans-perineal template biopsy,^37^ which might have affected the results (underestimate the specificity and overestimate the sensitivity) of the present study, it should be noted that TRUS biopsy is the accepted standard method in the current clinical practice and mostly used in biomarkers studies. Secondly, comparison with prostate biopsy pathology and not prostatectomy specimens is a similar limitation, possibly affecting the results in the same way, but prostate biopsy is the first approximation to diagnose and to stablish the risk category of the patients, so that it might represent more clearly the clinical practice. Thirdly, urine was collected with no prostate stimulation which could diminish the number of peptides specifically derived from the prostate secretion. Moreover, this study was performed retrospectively, however, on samples that were prospectively collected. Furthermore, the exact potential benefit for patients has to be assessed in a prospective trial. However, based on the data presented, implementation of this approach in an investigative setting appears highly justified.

The data presented in this study could demonstrate the utility of a multiple-marker approach for improved non-invasive detection of Sig PCa. Taking into consideration the increased variability which is caused by the high intra-tumour heterogeneity, an intrinsic characteristic of cancer, a single biomarker is not expected to enable the discrimination of Sig PCa from non-significant with high accuracy. Therefore, a combination of biomarkers appears to be the currently best option to guide biopsies and AS. Effective discrimination between clinically significant and non-significant PCa is expected to have a positive impact on reducing biopsies, improving patient compliance and also guide a more thorough examination in case of a positive result. The benefit for the management of patients under AS is also evident, as discrimination of the Sig PCa will result in improved guidance for initiation of definite treatment. Overall, improved non-invasive patient stratification is expected to present a positive impact on PCa patient management, by improving patient compliance and reducing over-treatment and the associated costs. The results of this study, although highly significant, will be assessed in a prospective trial to also determine the exact value in the context of patient management.

## Supplementary information

Supplementary Table 1

Supplementary Table 2

## Data Availability

All data generated or analysed during this study are included in this published article.
